# Supplementary material for: Does postoperative plasma IL-6 improve early prediction of infection after pulmonary cancer surgery? A two-centre prospective study
Source: PLoS One. 2025 Jun 23;20(6):e0326537. doi: 10.1371/journal.pone.0326537 (PMC12184903; doi:10.1371/journal.pone.0326537)
Supplement: S1 File — Patient enrolment, median perioperative biomarker concentrations, infection definitions, and univariable logistic regression analyses. (DOCX) [file pone.0326537.s001.docx]

**Supporting information**

**Patient enrolment.**


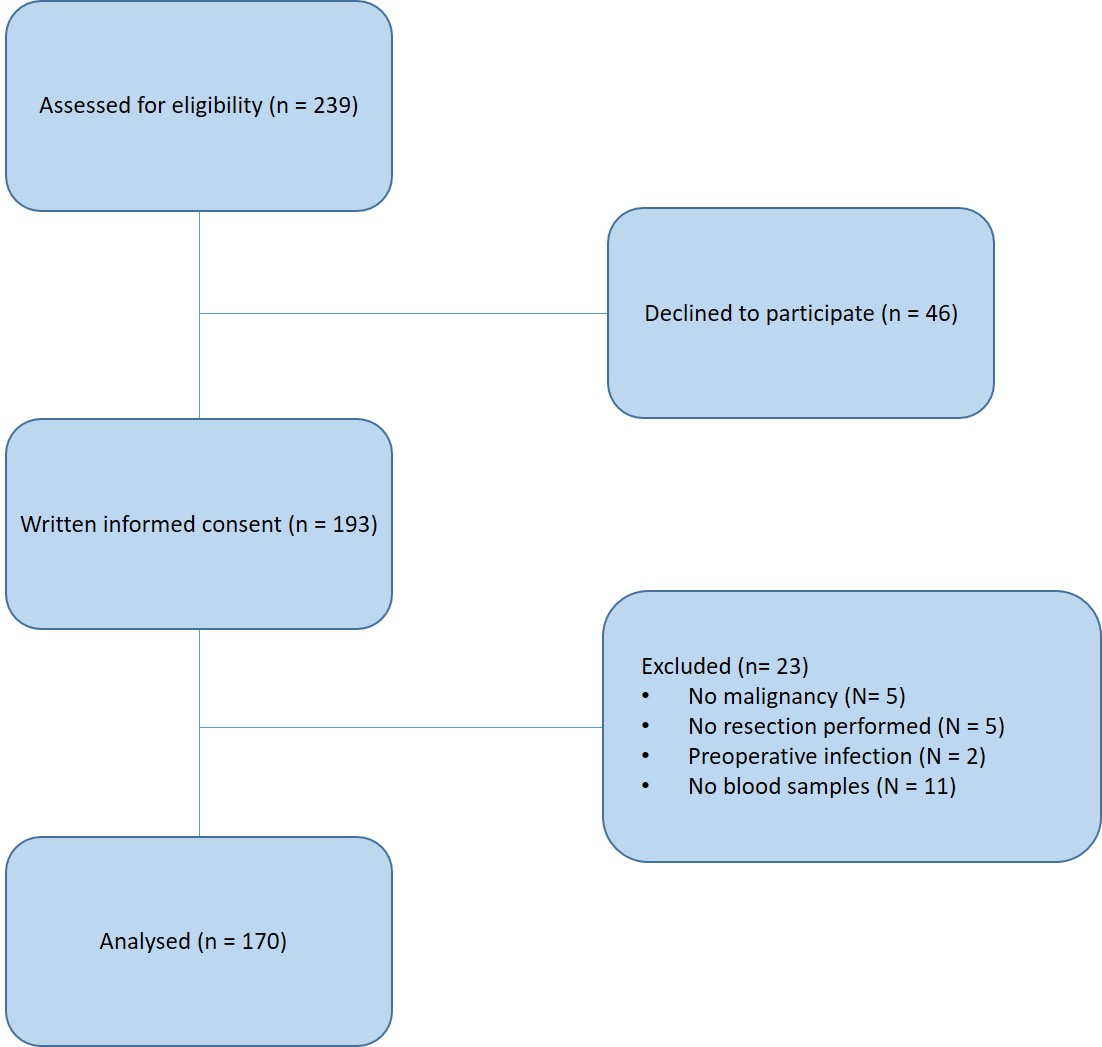


**Median perioperative biomarker concentrations.**

|  | **Infection** | **No infection** | **p-value** |
| --- | --- | --- | --- |
| **IL-6** |  |  |  |
| Preoperative | 6.14 (3.28, 10.88) | 3.40 (2.02, 5.98) | 0.002 |
| 6 hours | 123.00 (74.10, 170.00) | 83.10 (56.10, 133.00) | 0.029 |
| 9 hours | 68.00 (49.70, 122.00) | 51.95 (34.42, 74.08) | 0.010 |
| 12 hours | 67.90 (35.70, 117.00) | 38.50 (25.55, 61.15) | 0.003 |
| 24 hours | 37.80 (19.95, 85.22) | 25.10 (18.75, 40.88) | 0.057 |
| 48 hours | 66.10 (47.15, 158.00) | 34.20 (22.60, 60.10) | <0.001 |
| 72 hours | 60.40 (37.57, 145.75) | 25.50 (16.55, 48.25) | <0.001 |
| **CRP** |  |  |  |
| Preoperative | 3.40 (2.05, 22.05) | 1.75 (0.80, 6.03) | 0.016 |
| 6 hours | 5.00 (2.50, 24.20) | 2.80 (1.40, 10.20) | 0.027 |
| 9 hours | 13.50 (6.80, 30.20) | 7.65 (4.90, 15.70) | 0.016 |
| 12 hours | 28.30 (17.90, 48.90) | 19.25 (12.57, 27.02) | 0.003 |
| 24 hours | 64.75 (40.67, 89.47) | 45.85 (33.15, 61.98) | 0.004 |
| 48 hours | 88.70 (51.50, 143.15) | 54.20 (36.70, 83.23) | 0.001 |
| 72 hours | 123.00 (71.28, 169.60) | 62.30 (39.20, 105.00) | <0.001 |
| **WBC** |  |  |  |
| Preoperative | 6.75 (5.12, 8.90) | 6.10 (4.90, 7.60) | 0.174 |
| 6 hours | 14.15 (12.12, 17.00) | 14.45 (12.00, 16.80) | 0.825 |
| 9 hours | 14.05 (11.90, 16.62) | 14.25 (12.03, 16.40) | 0.929 |
| 12 hours | 13.10 (10.98, 15.45) | 13.05 (10.90, 15.20) | 0.959 |
| 24 hours | 12.10 (9.90, 14.30) | 12.55 (10.17, 14.40) | 0.581 |
| 48 hours | 11.65 (9.15, 13.78) | 10.30 (8.72, 12.07) | 0.150 |
| 72 hours | 10.45 (8.27, 13.23) | 8.80 (7.50, 11.05) | 0.017 |
| **PCT** |  |  |  |
| Preoperative | 0.05 (0.03, 0.07) | 0.05 (0.04, 0.07) | 0.709 |
| 6 hours | 0.07 (0.06, 0.11) | 0.07 (0.05, 0.11) | 0.376 |
| 9 hours | 0.13 (0.09, 0.23) | 0.15 (0.08, 0.25) | 0.878 |
| 12 hours | 0.17 (0.10, 0.25) | 0.21 (0.10, 0.38) | 0.504 |
| 24 hours | 0.20 (0.11, 0.33) | 0.22 (0.11, 0.39) | 0.937 |
| 48 hours | 0.19 (0.14, 0.26) | 0.20 (0.11, 0.34) | 0.9331 |
| 72 hours | 0.20 (0.13, 0.38) | 0.15 (0.10, 0.27) | 0.098 |

Median (IQR) biomarker concentrations on all time points stratified by postoperative infection, IL-6 = interleukin 6, CRP = C-reactive protein, WBC = white blood cell count, PCT = procalcitonin.

**Infection definitions.**

| **Type of infection** | **Definition** |
| --- | --- |
| Respiratory infection | Signs/Symptoms/Laboratory: ONE of (fever >38,0 C, WBC <4*10^9 or >12*10^9 L -1, altered mental status in >70 yr old with no other recognised cause) and TWO of (new onset purulent sputum/change in character of sputum/increased respiratory secretions or increased suctioning OR worsening cough/dyspnoea/tachypnoea OR rales or bronchial breath sounds OR worsening gas exchange), Imaging: two or more serial chest imaging results with either (new and persistent OR progressive and persistent) changes of (infiltrate OR consolidation OR cavitation) OR one of: - Organism seen on Gram stain of lung tissue or pleural fluid, or identification of pathogenic organism from fluid or tissue from affected site - Abscess or other evidence of infection on gross anatomical or histopathologic examination - Imaging test evidence of abscess or other infection which if equivocal is supported by clinical correlation, specifically, physician documentation of antimicrobial treatment for lung infection |
| Superficial surgical site infection | Involves only skin and subcutaneous tissue of the incision AND patient has at least one of the following: a, purulent drainage from the superficial incision, b, organism(s) identified from an aseptically-obtained specimen from the superficial incision or subcutaneous tissue by a culture or nonculture based microbiologic testing method which is performed for purposes of clinical diagnosis or treatment c, a superficial incision that is deliberately opened by a surgeon, physician or physician designee and culture or non-culture based testing of the superficial incision or subcutaneous tissue is not performed AND patient has at least one of the following signs or symptoms: localized pain or tenderness; localized swelling; erythema; or heat d, diagnosis of a superficial incisional SSI by a physician or physician designee |
| Deep surgical site infection | Involves deep soft tissues of the incision AND patient has at least one of the following: a, purulent drainage from the deep incision b, a deep incision that is deliberately opened or aspirated by a surgeon, physician or physician designee or spontaneously dehisces AND organism(s) identified from the deep soft tissues of the incision by a culture or non-culture based microbiologic testing method which is performed for purposes of clinical diagnosis or treatment or culture or nonculture based microbiologic testing method is not performed, A culture or non-culture based test from the deep soft tissues of the incision that has a negative finding does not meet this criterion, AND patient has at least one of the following signs or symptoms: fever (>38°C); localized pain or tenderness c, an abscess or other evidence of infection involving the deep incision detected on gross anatomical exam, histopathologic exam, or imaging test |
| Organ/ Space surgical site infection (including empyema) | Involves any part of the body deeper than the fascial/muscle layers that is opened or manipulated during the operative procedure AND patient has at least one of the following: a, purulent drainage from a drain placed into the organ/space b, organism(s) identified from fluid or tissue in the organ/space by a culture or non-culture based microbiologic testing method which is performed for purposes of clinical diagnosis or treatment c, an abscess or other evidence of infection involving the organ/space detected on: • gross anatomical exam or • histopathologic exam or • imaging test evidence definitive or equivocal for infection |
| Urinary system infection | One of: - Identification of pathogenic organism from fluid or tissue from affected site, - Abscess or other evidence of infection on gross anatomical examination, during invasive procedure, or during histopathologic examination, - ONE of Fever >38 C, localised pain or tenderness with no other recognised cause AND one of purulent drainage from affected site OR organism identified in blood by culture or non-culture based biological testing OR imaging suggestive of infection which if equivocal is supported by clinical correlation, specifically physician documented treatment for urinary system infection |

Definitions of infectious complications according to the definitions used by the Centers for Disease Control.

**Univariable logistic regression analyses on 30-day postoperative infection.**

| **Univariable Analysis** | | | |
| --- | --- | --- | --- |
| **Predictor** | **OR (95% CI)** | **C-statistic (95% CI)** | **p-value** |
| **IL-6 per 10 pg/ml** | 1.05 (1.00, 1.01) | 0.64 (0.53, 0.74) | 0.012 |
| **CRP per mg/L** | 1.02 (1.01, 1.03) | 0.66 (0.55, 0.76) | 0.004 |
| **PCT per ng/ml** | 1.29 (0.91, 1.82) | 0.49 (0.38, 0.60) | 0.156 |
| **WBC *10^9/L** | 1.03 (0.94, 1.12) | 0.50 (0.39, 0.61) | 0.518 |

Odds ratios (OR) and concordance statistic (c-statistic) with 95% Confidence Intervals (CI). IL-6 = Interleukin 6, CRP = C-reactive protein, PCT = procalcitonin, WBC = white blood cell count
