# Supplementary material for: Does postoperative plasma IL-6 improve early prediction of infection after pulmonary cancer surgery? A two-centre prospective study
Source: PLoS One. 2025 Jun 23;20(6):e0326537. doi: 10.1371/journal.pone.0326537 (PMC12184903; doi:10.1371/journal.pone.0326537)
Supplement: Attachment — Submitted filename: Review notes Plos ONE_IL-6.docx [file pone.0326537.s002.docx]

Review: **Does postoperative IL-6 improve early prediction of infection after pulmonary cancer**

**surgery? A two-centre prospective study**

**General Comments**

- Overall some of the findings of this paper are very important. Understanding the added value of IL-6 and CRP levels as an early predictor of infection after pulmonary cancer surgery is essential.
- The knowledge is useful for patient care and aid clinicians to minimize the risk of postoperative infection. Very good attempt by the authors.

**Section specific comments**

Title

- It could be worded to **Does postoperative serum or plasma IL-6 improve early prediction of infection after pulmonary cancer surgery? A two-centre prospective study**
- In the manuscript reference is made to blood sample but it’s not clear if plasma or serum was used. Using IL-6 could be misleading, a more specific matrix linked to the IL-6 levels would be appropriate, e.g. serum IL-6 or Plasma IL-6

Summary/Abstract

- Please clarify in the conclusion whether IL-6 is plasma or serum

Introduction

- It is good that the authors mentioned the well-established fact that IL-6 concentrations are elevated after surgery, trauma, and clinical illness.

Methods

- Study procedures and biomarker sampling – the authors mention “Blood samples were drawn from an arterial line directly after induction of general anaesthesia (further referred to as ‘preoperative sample’), and after 6, 9, 12, 24, 48, and 72 hours. Blood samples were

centrifuged and stored in a freezer at -80 °C and shipped to the clinical chemistry lab of the Sint Antonius hospital Nieuwegein for batch analysis.”

a. Can the authors specify what type of blood collection tubes, how many tubes and the volume of blood drawn?
b. Is it correct to assume that EDTA-blood was used for WBC measurements? Authors should provide clarity in the text.
c. What types of matrix was derived from the blood samples that were centrifuged and stored in a freezer at -80 °C? plasma or serum?
d. The goal was to determine if IL-6 and CRP levels observed within the first 24 hours after surgery are associated with postoperative infection risk. What is the justification of not including timepoints 0 and 3 hours?

Results

- In Table 1, there is significant difference for the surgery duration (in minutes) and blood loss between patients with and without postoperative infection. Considering that patients with infection had higher IL-6 levels, did the authors find any correction between the surgery time/intraoperative blood loss with the IL-6 levels?
- Since preoperative (Timepoint 0) and 3 hour postoperative levels of IL-6 were not presented in this study, how do the authors justify that Postoperative peak IL-6 concentrations occurred after 6 hours in the majority of patients?
- Base on the results, can the authors base their findings to IL-6 levels possibly reflect the severity of postoperative infection?

Discussion

- The general presentation in the discussion section looks ok.
- Considering the weight of gender(male) and age to their predictive performance model, the arguments could have benefitted from it although those facts are well established.
- Authors could consider the delta change in these biomarkers respective to the timepoints as a marker of postoperative infection risk, severity and/or recovery outcomes.

Figure 1

Preoperative (Time=0 hours) levels of the biomarkers are shown. Throughout the manuscript, there is no mention of this timepoint sampling. Where did this timepoint come from and were the levels indeed zero?
